# Clinical efficacy of combining fenofibrate with statins in patients with diabetes and hyperlipidemia: a meta-analysis

**DOI:** 10.3389/fendo.2026.1694928

**Published:** 2026-03-25

**Authors:** Jiayan Huang, Kaishun Meng, Huawei Qiu

**Affiliations:** 1 Department of Pharmacy, Guangdong Provincial Hospital of Chinese Medicine, Hainan Hospital (Hainan Traditional Chinese Medicine Hospital), Haikou, Hainan, China; 2 Department of Endocrinology, Sanya People’s Hospital, West China (Sanya) Hospital, Sichuan University, Sanya, Hainan, China

**Keywords:** diabetes mellitus, fenofibrate, hyperlipidemia, meta-analysis, statin

## Abstract

**Objective:**

To systematically assess the clinical effectiveness of combining fenofibrate with statins in treating patients with diabetes mellitus and hyperlipidemia.

**Methods:**

Clinical randomized controlled trials assessing the efficacy of fenofibrate and statins in patients with diabetes mellitus and hyperlipidemia were identified from both Chinese and international databases. The experimental group received fenofibrate combined with statins, while the control group received either statins or fenofibrate alone, statins or fenofibrate with placebo, placebo alone, or lifestyle interventions.

**Results:**

The analysis incorporated 18 randomized controlled trials with a combined participant count of 2113. The analysis showed that patients in the experimental group had a higher overall efficacy rate than those in the control group (OR = 5.42, 95% CI = 3.11 to 9.45, P < 0. 00001). Furthermore, levels of total cholesterol (SMD = -1.01, 95% CI = -1.60 to -0.41, P = 0.0009), high-density lipoprotein cholesterol (SMD = 1.31, 95% CI = 0.86 to 1.76, P < 0.00001), triglycerides (SMD = -0.94, 95% CI = -1.59 to - 0.30, P = 0. 004), low-density lipoprotein cholesterol (SMD = -2.26, 95% CI = -3.05 to -1.47, P < 0. 00001), fasting plasma glucose (SMD = -0.37, 95% CI = -0.51∼-0.23, P<0. 00001), and postchallenge plasma glucose (SMD =-0.88, 95% CI =-1.19∼-0.57, P<0. 00001) showed significant improvements compared to those in the control group. All of the above differences were statistically significant.

**Conclusion:**

This meta-analysis shows that the combination of fenofibrate and statins is superior to statin monotherapy in improving lipid and glycemic profiles (surrogate markers) in patients with diabetes and hyperlipidemia. However, its long-term benefits on cardiovascular hard endpoints need further confirmation, and combination therapy may increase the risk of adverse reactions such as muscular and hepatic events. Clinical application requires balancing benefits and risks, accompanied by enhanced monitoring.

## Introduction

Most diabetes mellitus patients exhibit hyperlipidemia (HLP), an atherogenic lipid disorder marked by increased small, dense low-density lipoprotein cholesterol (LDL-C) particles, elevated triglycerides (TG), and decreased high-density lipoprotein cholesterol (HDL-C) levels (1). Elevated LDL-C and enhanced glycosylation of low-density lipoprotein (LDL) heighten the risk of atherosclerosis in patients with diabetes mellitus and hyperlipidemia, potentially impacting quality of life and patient safety. In this context, statins or fibrates may be utilized, with statins demonstrating a reduction in atherosclerosis-related morbidity and mortality among diabetic patients (2, 3). Fibrates lower triglyceride levels and raise HDL-C, which may enhance lipid-related cardiovascular risk indicators (4, 5); however, randomized controlled trials have not consistently shown their impact on reducing cardiovascular morbidity and mortality. The American Diabetes Association (ADA) (6) suggests that statin or fibrate monotherapy impacts various aspects of lipoprotein metabolism, posing challenges for altering the lipid profile in patients with diabetes and hyperlipoproteinemia (HLP). Given the complementary effects of statins (potently lowering LDL-C) and fibrates (significantly improving TG/HDL-C dysregulation), most patients with diabetes and hyperlipidemia require combination therapy to achieve comprehensive lipid control (7). Recent studies increasingly examine the combination of fenofibrate and statins, yet their clinical efficacy remains debated. This study aims to meta-analyze the clinical efficacy of combining fenofibrate with statins for treating diabetes mellitus with hyperlipidemia, providing a reference for this treatment approach.

## Methods

### Inclusion and exclusion criteria

Inclusion criteria: (1) Study design: Only randomized controlled trials (RCTs) were considered, regardless of blinding procedures, with publications restricted to Chinese and English. (2) Participants: Individuals with a confirmed diagnosis of both diabetes mellitus and hyperlipidemia were included; (3) Interventions: Patients in the experimental group received a combination of fenofibrate and statins without restrictions on type, manufacturer, dosage form, or specification. In contrast, the control group was administered either statins or fenofibrate, statins with a placebo, fenofibrate with a placebo, a placebo alone, or lifestyle interventions. (4) Outcome indicators: HDL-C level, Total Cholesterol (TC) level, TG level, LDL-C level, Fasting Plasma Glucose (FPG), Postchallenge Plasma Glucose (PPG), and overall efficacy rate (OER), Included studies should contain at least one of the above outcome indicators.

Exclusion criteria include studies lacking sufficient data, duplicate publications, inaccessible full texts, non-randomized controlled trials, reviews, conference abstracts, case reports, and animal studies.

### Search strategy

A comprehensive search was conducted across multiple databases, including China National Knowledge Internet (CNKI), Wanfang, Vip, China Biomedical Literature Database, PubMed, Cochrane Library, Embase, and Web of Science, all from inception to November 2024. The search terms were: “Fenofibrate”, “Hydroxymethylglutaryl-CoA Reductase Inhibitors” OR “Statins” OR “atorvastatin” OR “simvastatin” OR “rosuvastatin” OR “pravastatin” OR “lovastatin” OR “fluvastatin” OR “pitavastatin”, “Hyperlipidemias” OR “Lipidemia” OR “Hyperlipemia”, “Diabetes Mellitus”. A combination of subject terms and free words was used to simultaneously search for references incorporated into the literature. The search strategy was exemplified by pubmed: (((Fenofibrate[MeSH Terms]) AND (((((((((Hydroxymethylglutaryl-CoA Reductase Inhibitors[MeSH Terms]) OR (Statins[Title/Abstract])) OR (atorvastatin[Title/Abstract])) OR (simvastatin[Title/Abstract])) OR (rosuvastatin[Title/Abstract])) OR (pravastatin[Title/Abstract])) OR (lovastatin[Title/Abstract])) OR (fluvastatin[Title/Abstract])) OR (pitavastatin[Title/Abstract]))) AND (((Hyperlipidemias[MeSH Terms]) OR (Lipidemia[Title/Abstract])) OR (Hyperlipemia[Title/Abstract]))) AND (Diabetes Mellitus[MeSH Terms]).

### Screening of literature and extraction of data

Literature screening and data extraction were performed independently by two researchers (Huang and Meng). First, all retrieved records were imported into the NoteExpress reference management software for automatic deduplication. Subsequently, the two researchers independently reviewed the titles and abstracts of the remaining records for initial screening based on the inclusion and exclusion criteria. Full texts of records that passed the initial screening were then obtained and reviewed to determine final inclusion. Any disagreements during the screening process were resolved through discussion between the two researchers, with arbitration by a third researcher if consensus could not be reached.

A pre-designed, standardized data extraction form was used. Extracted information included: first author, publication year, country, study design, sample size (intervention/control groups), patient baseline characteristics (age, sex, types of diabetes and hyperlipidemia), details of interventions (drug types, doses, routes of administration for both the experimental and control groups), treatment duration, outcome data (mean values, standard deviations, or convertible data for each indicator; number of events for overall effective rate), follow-up time, and reports of adverse reactions (if reported). For studies with incomplete data, we attempted to contact the original authors via email to obtain the information. All extracted data were cross-checked by a third researcher to ensure accuracy.

### Quality evaluation of literature

The methodological quality of the included randomized controlled trials was independently assessed by two researchers using the Cochrane Collaboration’s ‘Risk of Bias’ tool (8). This tool evaluates the risk of bias across seven domains: (1) random sequence generation (selection bias); (2) allocation concealment (selection bias); (3) blinding of participants and personnel (performance bias); (4) blinding of outcome assessment (detection bias); (5) incomplete outcome data (attrition bias); (6) selective reporting (reporting bias); and (7) other potential biases. Judgments for each domain were categorized as ‘low risk’, ‘high risk’, or ‘unclear risk’. The assessment results from the two researchers were reconciled through discussion, with arbitration by a third researcher in case of disagreement. A final overall risk of bias graph was generated using RevMan 5.2 software.

### Statistical methods

The statistical analysis of clinical heterogeneity was performed using RevMan5.2 software, employing the I² test. If the results showed P ≥ 0.05 and I² < 50%, it suggested minimal heterogeneity across studies, prompting the application of a fixed-effect model for Meta-analysis. In cases where significant heterogeneity was detected (P < 0.05 or I² ≥ 50%), potential sources of variation were investigated. If no clear clinical or methodological differences were identified, a random-effects model was applied. For continuous variables such as lipid and blood glucose levels, the standardized mean difference (SMD) along with its 95% confidence interval (CI) served as the effect measure. Meanwhile, dichotomous outcomes, like occurrence rates, were evaluated using odds ratios (OR) with 95% CIs. A P-value below 0.05 was considered statistically significant. Given the variations in the types and doses of statins used across the included studies, this was considered a potentially important source of clinical heterogeneity and was taken into account when interpreting the results.

## Results

### Basic information of included studies and quality evaluation results

**Figure 1:**
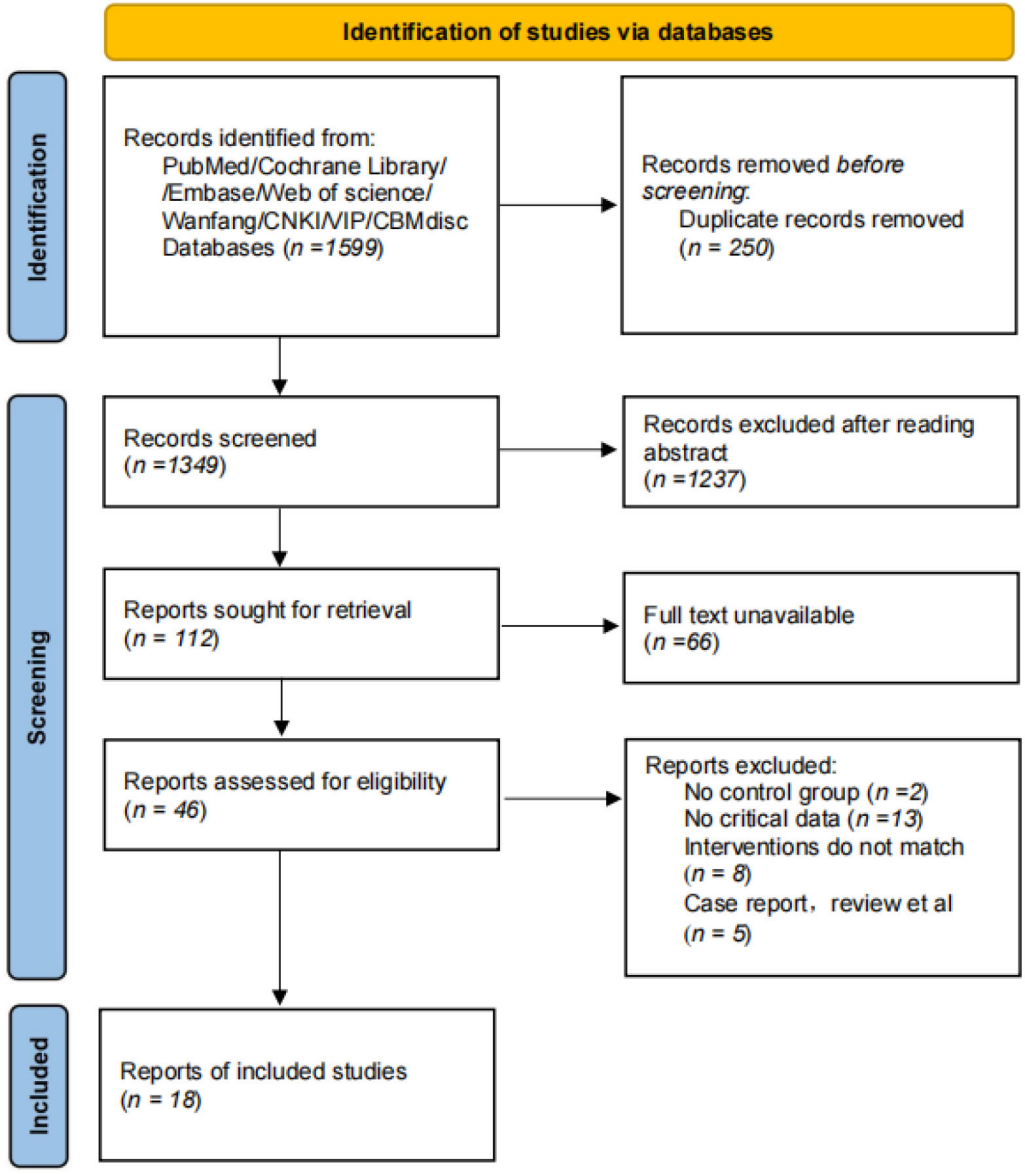
Flow chart of literature screening.

The database search yielded 1599 documents, with 18 ultimately included (9–26). As shown in **Figure 1**, the steps for filtering the literature are outlined.

### Basic characteristics and the results of quality evaluation

**Figure 2:**
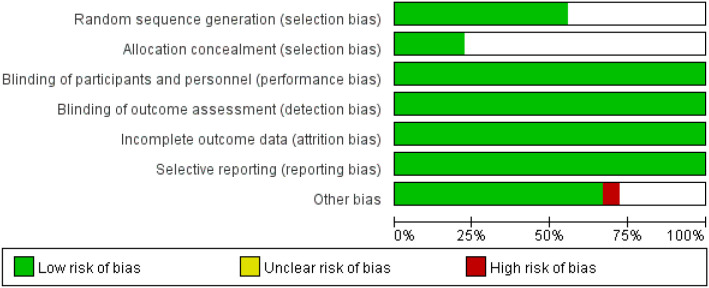
Risk of bias assessment diagram of the included literature.

There was a total of 18 included literatures (9–26), 6 English literatures and 12 Chinese literatures. The study included 2113 patients with both diabetes mellitus and hyperlipidemia, with 1061 in the intervention group and 1052 in the control group. **Table 1** presents the fundamental attributes of the literature. The evaluation of the included literature revealed 4 A-grade and 14 B-grade studies. The risk of bias assessment diagram of the included literature is shown in **Figure 2**.

**Table 1:** Basic characteristics of the included literature.

Author and year	Country	Sample size	Age		Intervention programs		Duration of treatment	Assessment tools
			T	C	Experimental group	Control group		
Xiong 2006 (9)	China	T:40 C:40	53.0 ± 10.2	55.0 ± 9.0	Simvastatin 10mg+Fenofibrate 200mg	Simvastatin 20mg	6 months	①②③④
Farnier2011 (10)	many countries	T:146 C:145	56.1 ± 8.3	57.2 ± 9.5	Pravastatin 40mg+Fenofibrate 160mg	Simvastatin 20mg	12 weeks	①②③④
Farnier2012 (11)	many countries	T:135 C:137	61.7 ± 8.4	60.6 ± 7.5	Pravastatin 40mg+Fenofibrate 160mg+Ezetimibe 10mg	Simvastatin 20mg +Ezetimibe 10mg	12 weeks	①②③④
Wang 2021 (12)	China	T:75 C:75	59.7 ± 2.8	58.5 ± 2.2	Fluvastatin 40mg+Fenofibrate 10mg	Fluvastatin 40mg	2 months	①②③④⑤⑥
Derosa2004 (13)	Italy	T:25 C:23	61 ± 5	59 ± 6	Fluvastatin 80mg+Fenofibrate 200 Mg	Fluvastatin 80 Mg	12 months	①②③④⑤⑥
Durrington 2004 (14)	many countries	T:60 C:51	–	–	Rosuvastatin 5mg/Fenofibrate 67mg	Rosuvastatin 10 Mg	24 weeks	①②③④⑦
Athyros2002 (15)	USA	T:40 C:40	–	–	Atorvastatin 20mg+Fenofibrate 200mg	Atorvastatin 20mg	24 weeks	①②③④⑦
Lella2013 (16)	Netherlands	T:28 C:30	51.33 ± 9.5	48.16 ± 8.4	Atorvastatin 10mg+Fenofibrate 145mg	Atorvastatin 10mg	12 weeks	①②③④
Li2019 (17)	China	T:54 C:54	55.61 ± 10.37	52.61 ± 10.21	Fluvastatin 20mg+Fenofibrate 200mg	Fluvastatin 20mg	10 weeks	①②③④⑤⑥⑦
Sun 2017 (18)	China	T:40 C:40	39.5 ± 3.6	41.7 ± 3.8	Rosuvastatin 10mg+Fenofibrate 200mg	Rosuvastatin 10mg	12 weeks	①②③④⑤⑥⑦
Chen2017 (19)	China	T:57 C:57	57.00 ± 12.00	56.75 ± 11.25	Fluvastatin 40mg+Fenofibrate 200mg	Fluvastatin 40mg	6 months	①②③④⑤⑥⑦
Liao 2014 (20)	China	T:48 C:48	–	–	Fluvastatin 40mg+Fenofibrate 200mg	Fluvastatin 40mg	6 months	①②③④⑤⑥⑦
Xia 2014 (21)	China	T:30 C:30	–	–	Fluvastatin 40mg+Fenofibrate 200mg	Fluvastatin 40mg	8 weeks	①②③④⑤⑥⑦
Zhang 2012 (22)	China	T:106 C:105	–	–	Fluvastatin 40mg+Fenofibrate 200mg	Fluvastatin 40mg	8 weeks	①②③④⑤⑥
Liu 2007 (23)	China	T:45 C:45	–	–	Pravastatin 10mg+Fenofibrate 200mg	Pravastatin 10mg	12 weeks	①②③④
Tai 2021 (24)	China	T:65 C:65	62.5 ± 4.3	62.0 ± 4.5	Atorvastatin 20mg+Fenofibrate 200mg	Atorvastatin 20mg	12 weeks	①②③④⑤⑥⑦
Zhang 2014 (25)	China	T:37 C:37	47.3 ± 6.1	48.2 ± 7.8	Simvastatin Tablets 10mg+Fenofibrate 250mg	Simvastat intablets 10mg	8 weeks	①②③④
Guo 2011 (26)	China	T:30 C:30	–	–	Simvastatin20 mg+Fenofibrate 200mg	Simvastatin 40mg	8weeks	①②③④

T is the experimental group, C is the control group, ① is the LDL-C level, ② is the TC level, ③ is the TG level, ④ is the HDL-C level, ⑤ is the FPG, ⑥ is the PPG, and ⑦ is the OER.

### Meta-analysis results

**Figure 3:**
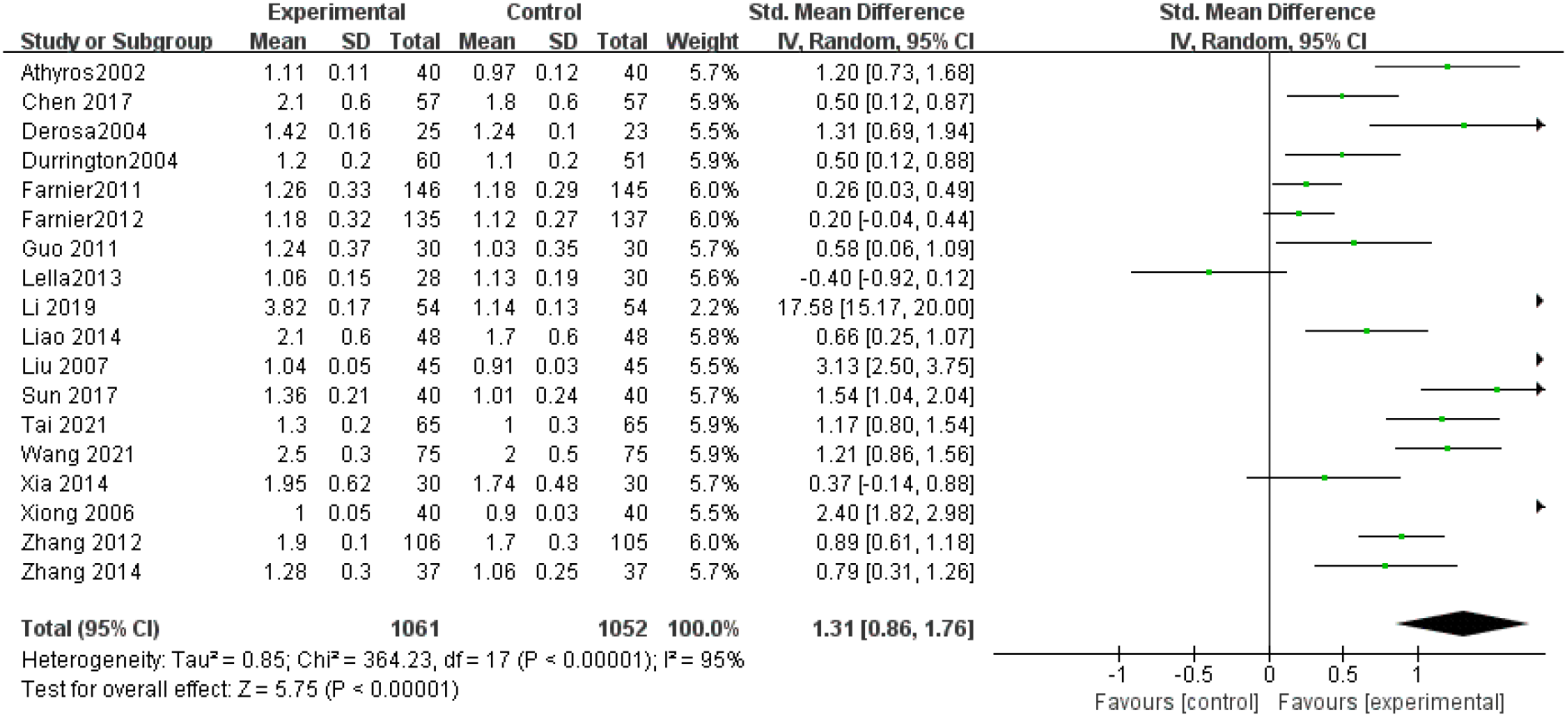
Forest plot of meta-analysis of HDL-C levels.

HDL-C levels: A total of 18 research articles (9–26) assessed HDL-C concentrations, demonstrating notable variability (P < 0.00001). Given the substantial heterogeneity (I² = 95%), a random-effects approach was employed after sensitivity testing. The results revealed that HDL-C levels were markedly elevated in the experimental group relative to controls (SMD = 1.31, 95% CI: 0.86–1.76, P < 0.00001), as illustrated in **Figure 3**.

**Figure 4:**
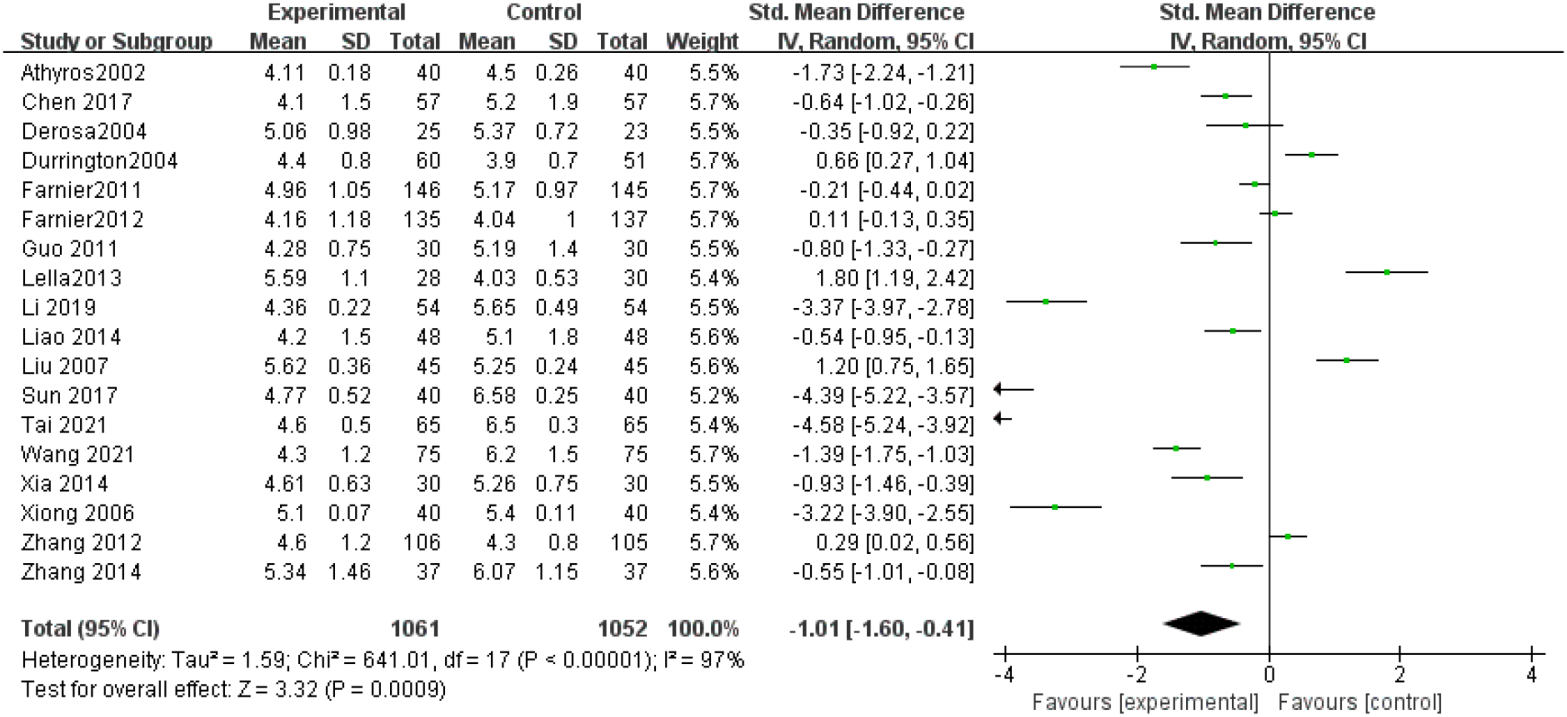
Forest plot of meta-analysis of TC levels.

TC levels: A total of 18 studies (9–26) evaluated TC concentrations, demonstrating significant variation (P < 0.00001). Because of the considerable heterogeneity (I² = 97%), sensitivity analysis led to the use of a random effects model. The findings revealed markedly lower TC levels in the experimental group versus the control (SMD = -1.01, 95% CI = -1.60 to -0.41, P = 0.0009), illustrated in **Figure 4** (p = 0.0009).

**Figure 5:**
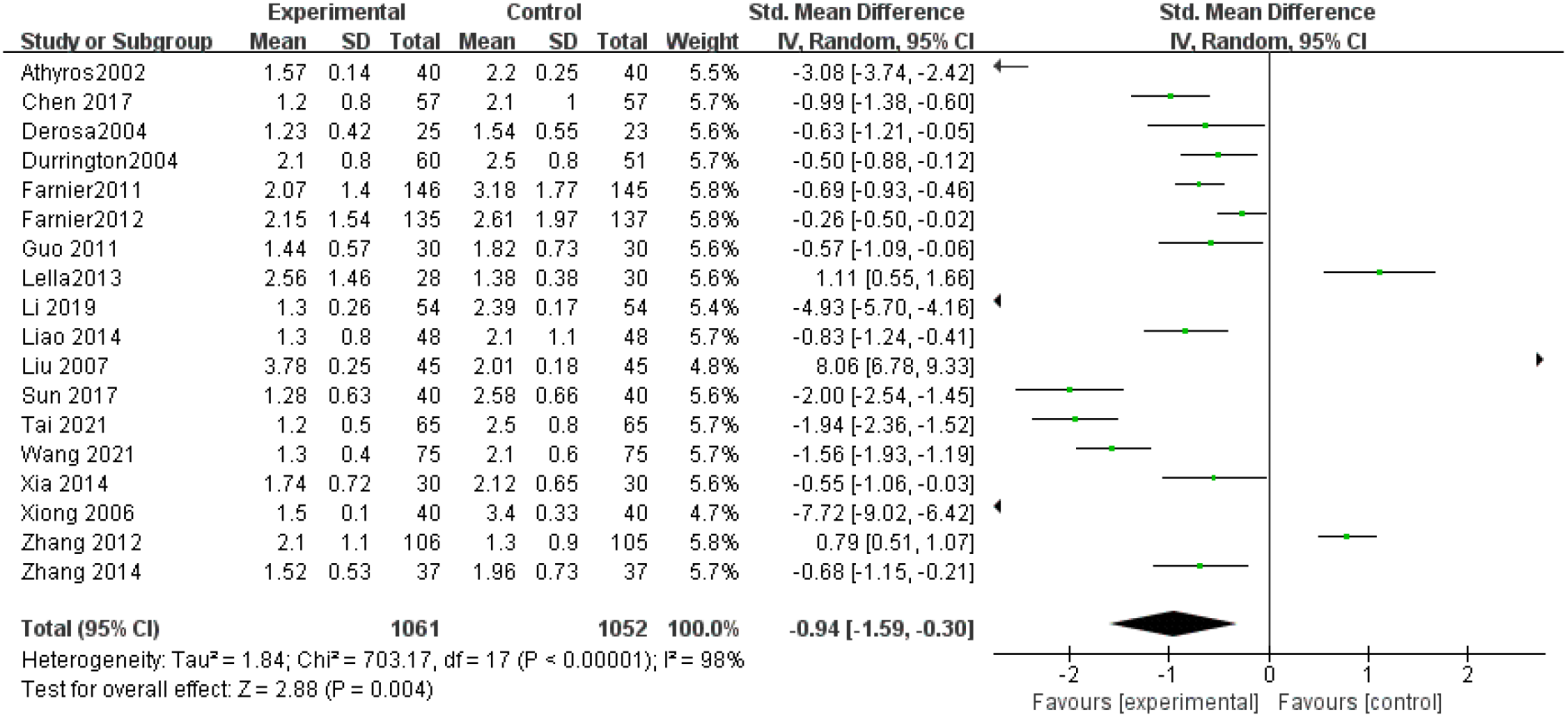
Forest plot of meta-analysis of TG levels.

TG levels: A total of 18 research articles (9–26) investigated TG levels, demonstrating substantial heterogeneity (P < 0.00001). Due to the high inconsistency (I² = 98%), a random-effects model was employed after sensitivity assessment. The analysis indicated that the experimental group had markedly reduced TG levels relative to the control group (SMD = -0.94, 95% CI: -1.59 to -0.30, P = 0. 004), as illustrated in **Figure 5**.

**Figure 6:**
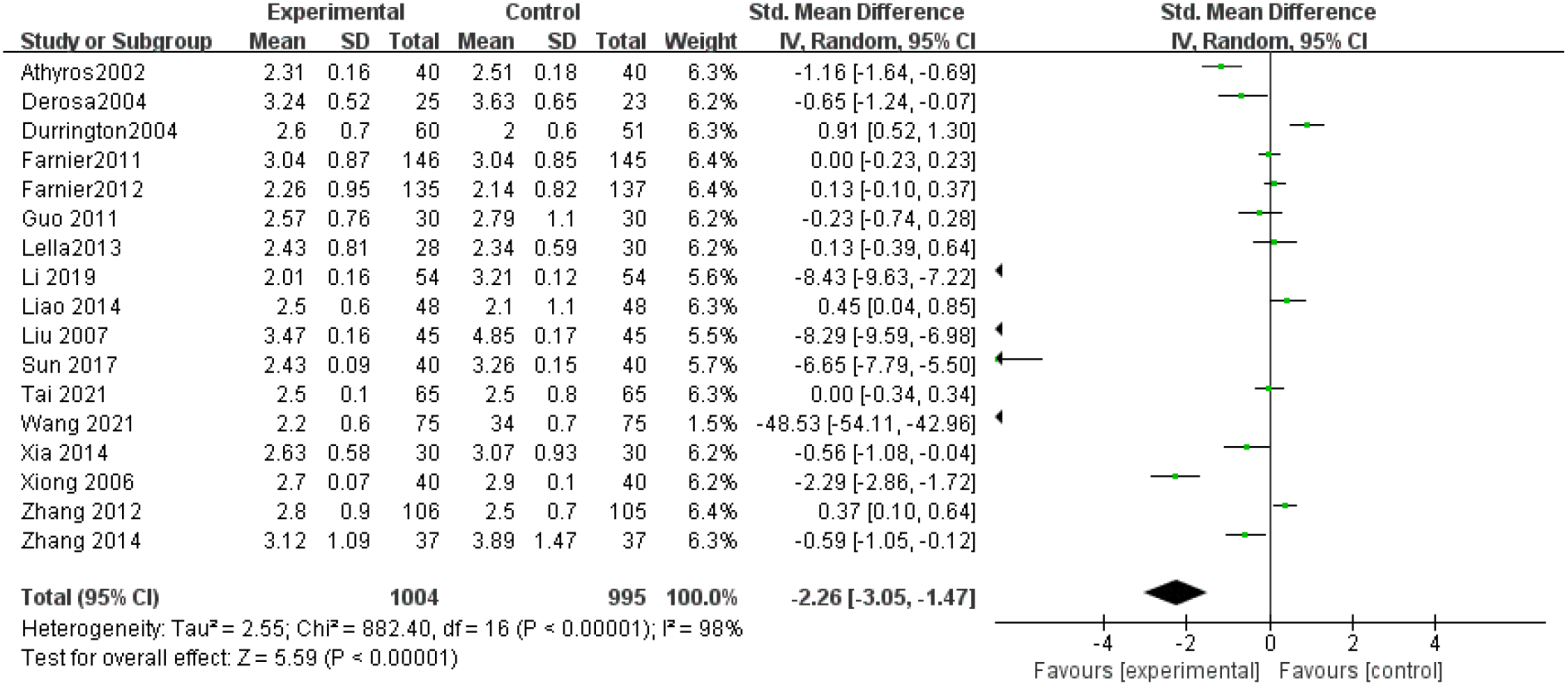
Forest plot of meta-analysis of LDL-C levels.

LDL-C levels: Among the included research, seventeen trials (9–18, 20–26) measured LDL-C levels, showing considerable heterogeneity (P < 0.00001, I^2^ = 98%). Due to the high inconsistency (I² = 98%), a random-effects model was employed after sensitivity analysis. The results demonstrated a notable decrease in LDL-C levels in the intervention group relative to controls (SMD = -2.26, 95% CI: -3.05 to -1.47, P < 0.00001), as illustrated in **Figure 6**.

**Figure 7:**
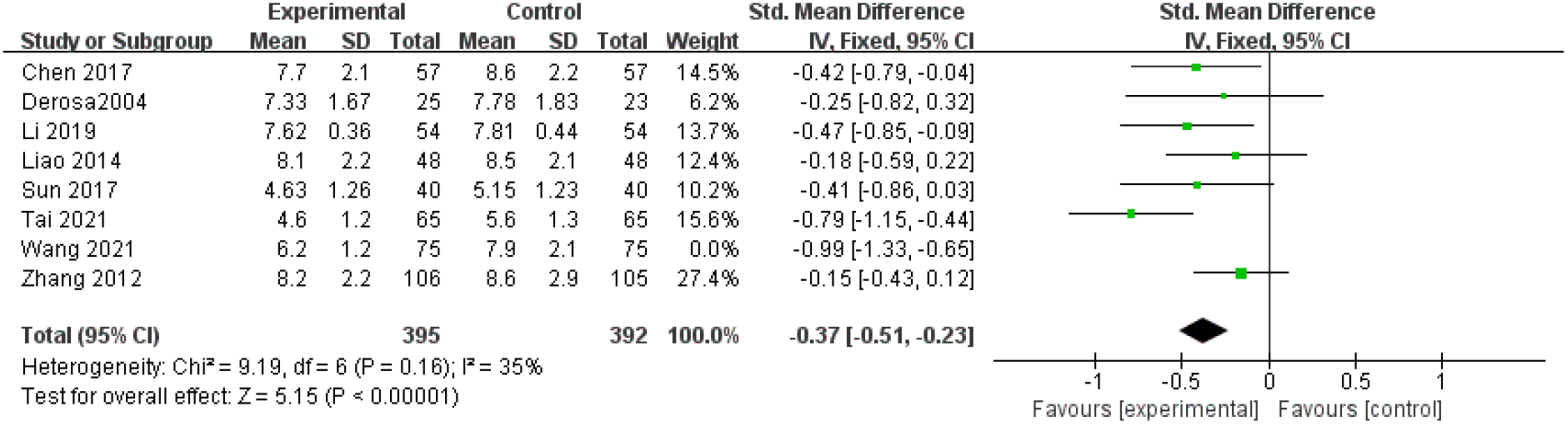
Forest plot of meta-analysis of FPG levels.

FPG levels: Eight studies (12, 13, 17–20, 22, 24) reported fasting plasma glucose (FPG) levels, exhibiting significant heterogeneity (P = 0.006, I^2^ = 65%). After excluding Xiaoni Wang’s study (12) through sensitivity analysis, heterogeneity among studies decreased (P = 0.16, I^2^ = 35%), consistently employing a fixed-effect model. The findings indicated a statistically significant reduction in FPG levels in the experimental group compared to the control group (SMD = -0.37, 95% CI = -0.51 to -0.23, P < 0.00001), as shown in **Figure 7**.

**Figure 8:**
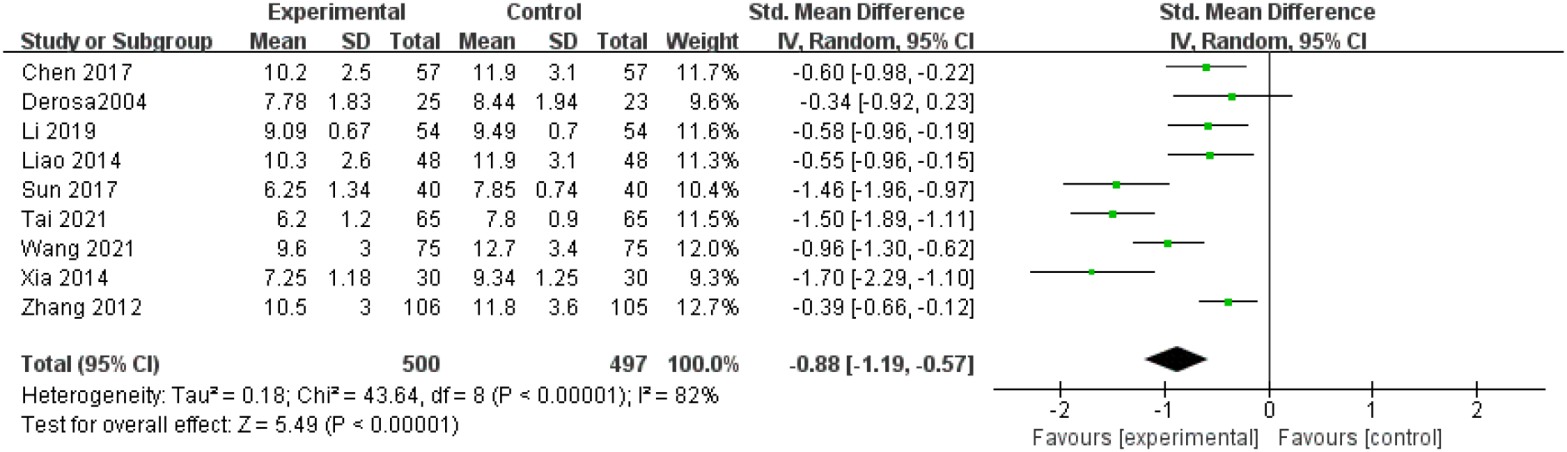
Forest plot of meta-analysis of PPG levels.

PPG levels: Nine studies (12, 13, 17–22, 24) reported PPG levels, revealing significant heterogeneity (P < 0.00001, I^2^ = 82%). A random effects model was applied after conducting a sensitivity analysis. The results showed that that patients in the experimental group exhibited significantly lower PPG levels compared to the control group (SMD = -0.88, 95% CI = -1.19 to -0.57, P < 0.00001), as shown in **Figure 8**.

**Figure 9:**
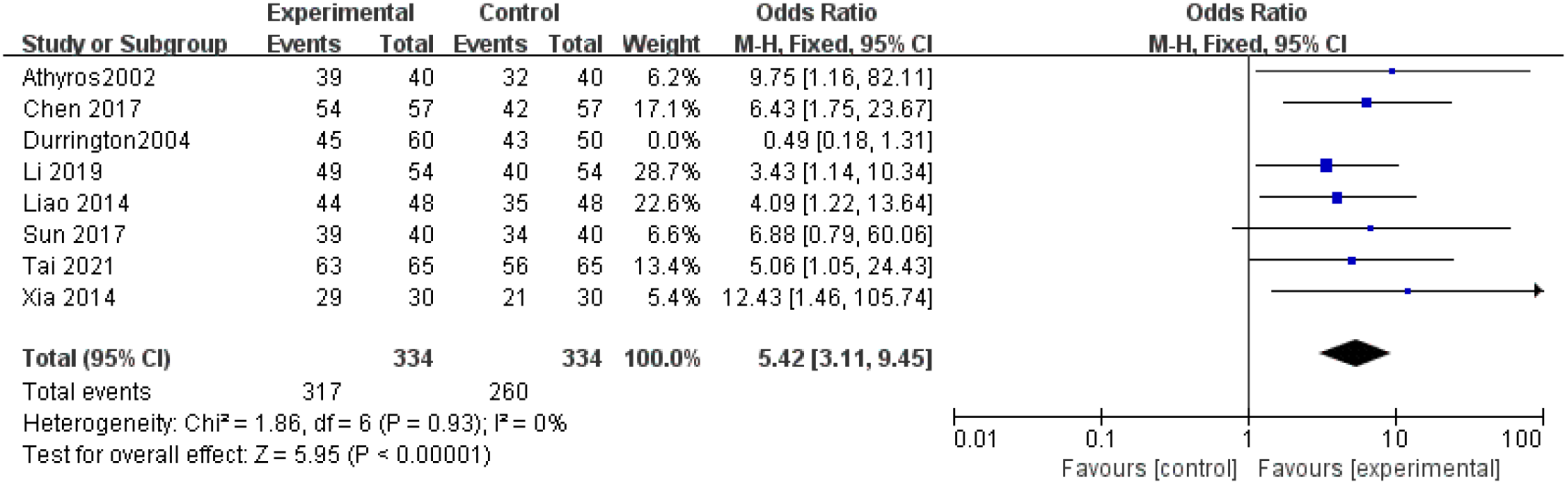
Forest plot of meta-analysis of OER.

OER: Eight studies (14, 15, 17–21, 24) examined the OER, revealing significant heterogeneity (P = 0.001, I^2^ = 62%), and no heterogeneity among the studies after excluding Durrington’s study (14) by a sensitivity analysis (P = 0.93, I^2^ = 0%), inherently using a fixed-effects model. The findings indicated a statistically significant higher OER in the experimental group compared to the control group (OR = 5.42, 95% CI = 3.11 to 9.45, P < 0.00001), as shown in **Figure 9**.

## Discussion

The findings of this meta-analysis regarding the efficacy of combination therapy should be interpreted within the context of two key clinical considerations. First, while significant improvements were observed in surrogate markers such as lipid and glycemic profiles, current evidence from large randomized controlled trials regarding the benefits of fibrates (including fenofibrate) on major adverse cardiovascular events (such as myocardial infarction, cerebrovascular events, and cardiovascular mortality) as hard endpoints in patients with diabetes and hyperlipidemia remains inconsistent or insufficient. This suggests that the long-term cardiovascular outcome benefits of combination therapy should be interpreted with caution and require validation through future large-scale studies targeting hard endpoints. Second, the safety of combination therapy is a core clinical concern that cannot be overlooked. This meta-analysis could not perform a quantitative synthesis of adverse reactions due to inconsistent reporting in the original data; however, the safety of combination therapy is a core clinical concern that cannot be overlooked. The combination of statins and fenofibrate may increase the risk of specific adverse events. The primary risk is muscle-related events, including myalgia, myositis, and the rare but serious rhabdomyolysis. The mechanism may be related to the interaction between the two drugs, as both can cause myopathy and are metabolized via the cytochrome P450 system, particularly statins metabolized by CYP3A4 (27). The second concern is hepatic impact, as both drugs can elevate serum transaminases. Additionally, fenofibrate is primarily excreted renally; therefore, dose adjustment and caution are required in patients with renal insufficiency to avoid accumulation toxicity (4). Consequently, before initiating combination therapy in clinical practice, baseline hepatic and renal function as well as creatine kinase levels should be assessed. Regular monitoring during treatment is recommended (e.g., 4–8 weeks after initiation, followed by periodic checks), and patients should be informed to promptly report unexplained muscle pain, tenderness, weakness, or dark urine. The risk may be increased in elderly patients, those with low body weight, multiple comorbidities, or on multiple concomitant medications, warranting extra caution. Overall, combination therapy can be used safely with strict patient selection and close monitoring, but its risk-benefit ratio requires individualized assessment for each patient.

This study demonstrated that combining fenofibrate with statins is more effective than using statins alone for treating patients with diabetes mellitus and hyperlipidemia. In patients with diabetes mellitus and hyperlipidemia, combining fenofibrate with treatment significantly reduced TC, TG, LDL-C, FPG, and PPG levels, while increasing HDL-C levels and OER, compared to statins alone (P < 0.05).

This study demonstrated that combining fenofibrate with statins is more effective than statin monotherapy in treating patients with diabetes mellitus and hyperlipidemia, significantly reducing TC, TG, LDL-C, FPG, and PPG levels while increasing HDL-C levels and OER (P < 0.05). The addition of fenofibrate contributes critically to the reduction of triglyceride (TG) levels. These findings align with those of JOSE et al. (28), whose study also indicated that for patients with type 2 diabetes mellitus and HLP, combining a statin with fenofibrate was more effective than monotherapy in normalizing lipid profiles while maintaining an acceptable safety profile. From the perspective of mechanism of action, the benefit of combination therapy stems from the complementary effects of the two drugs. Statins primarily inhibit cholesterol synthesis in hepatocytes, significantly reducing LDL-C and TC, with the magnitude of reduction closely related to their type and dose. The substantial decrease in LDL-C (SMD = -2.26) observed in this study primarily reflects the effect of the specific statins used in the combination. Fenofibrate, as a peroxisome proliferator-activated receptor alpha agonist, primarily activates lipoprotein lipase, thereby potently reducing TG and increasing HDL-C. Therefore, the combination therapy can simultaneously address different aspects of dyslipidemia in patients with diabetes and hyperlipidemia, achieving more comprehensive lipid management. The variability in the types and doses of statins across the studies included in this meta-analysis is also a potential reason for the high heterogeneity observed in various outcome measures.

This study found that combining statins with fenofibrate was more effective in reducing FPG and PPG than using statins alone, aligning with Papadopoulos et al. (29). Numerous studies (30, 31) have established that diabetes significantly increases the risk of cardiovascular disease and other complications, while TG serve as an independent risk factor for type 2 diabetes. Lipid and glucose metabolism affect each other for a long time, causing metabolic disorders in the body. Fenofibrate effectively reduces blood lipids and improves glucose levels, indicating that combining statins with fenofibrate may benefit patients with diabetes mellitus and hyperlipidemia by lowering both blood lipids and glucose.

This Meta-analysis faced limitations as some included RCTs lacked clarity on blinding, allocation concealment, and other details, resulting in overall low-quality literature. The dosage and duration of the drugs used in the studies were inconsistent; the available studies included a wide range of statins without in-depth categorization; due to the limited amount of data extracted, our study was not analyzed in terms of drug safety aspects and adverse effects.

In summary, combining statins with fenofibrate is more effective for treating diabetes mellitus with hyperlipidemia than using statins alone. Nonetheless, further validation through high-quality, large-sample randomized controlled trials is necessary for these meta-analysis findings.

## Data Availability

The original contributions presented in the study are included in the article/supplementary material. Further inquiries can be directed to the corresponding author.
